# Evaluation of a school-based HIV prevention intervention among Yemeni adolescents

**DOI:** 10.1186/1471-2458-11-279

**Published:** 2011-05-07

**Authors:** Buthaina Al-Iryani, Huda Basaleem, Khaled Al-Sakkaf, Rik Crutzen, Gerjo Kok, Bart van den Borne

**Affiliations:** 1 Department of Health Promotion, Faculty of Medicine, Health, and Life-Sciences, Maastricht University, the Netherlands; 2 United Nations Children's Fund, Sana'a, Yemen; 3 Department of Public Health and Community Medicine, Faculty of Medicine and Health Sciences, Aden University, Republic of Yemen; 4 Department of Work and Social Psychology, Faculty of Psychology, Maastricht University, the Netherlands

## Abstract

**Background:**

This article describes an evaluation of a school-based peer education intervention for HIV prevention among students in twenty seven high schools in Aden, Yemen. The intervention was developed after a survey among the same population in 2005, which revealed a high level of stigma towards people living with HIV (PLWH) and a low level of HIV knowledge.

**Methods:**

In a quasi-experimental design students who received the peer education intervention (78.6%) were compared with students who did not receive the intervention (21.4%). No systematic procedure was applied in selecting students for the intervention condition. Data were collected using a self-administered questionnaire from a sample of 2510 students from all 27 high-schools in Aden governorate. To increase internal validity, students were also compared with a cohort control sample surveyed in 2005, which was a random sample of 2274 students from the same schools.

**Results:**

Sixty eight percent of students targeted by peer education had good knowledge scores, compared with 43.3% of students not targeted by peer education (χ^2 ^= (df = 1) = 111.15, p < .01). Multi-level regression analysis revealed that, although there was a significant difference among schools, the intervention effect of peer education at the individual level was significant; students who received peer education had a statistically higher knowledge score(9.24 out of 12.0) compared with those not targeted (7.89 out of 12.0), OR = 2.11, 95% CI = 1.04-4.27, p < .05). Compared with the 2005 cohort control sample, students targeted by peer education had better knowledge on the modes of transmission and prevention and fewer misconceptions; and knowledge on the use of condoms increased from 49.4% to 67.8%. In addition, students who received the peer education interventions suggested significantly more actions to provide care and support for PLWH. Also, the levels of stigma and discrimination were much higher among the 2005 cohort control group, compared with those who received the peer education intervention.

**Conclusion:**

The school-based peer education intervention has succeeded in improving levels of knowledge on modes of transmission and prevention, and in decreasing levels of stigma and discrimination in a culturally conservative setting.

## Background

Yemen is one of the poorest countries in the Middle East and North Africa (MENA) region [1], and one of the poorest countries in the world, where more than 45% of the population lives in poverty [2]. Yemen's population is around 22.5 million people; and at 3.02%, the country has one of the highest population growth rates globally, with the population expected to double in the next 23 years[3]. The population in Yemen is one of the youngest in the MENA region, and unlike many countries in the region where the youth bulge has already peaked, the share of youth in Yemen's total population will not begin to diminish for many years to come[1]. The Yemeni economy is caught in a slow growth cycle, leading to stagnant per capita incomes and rising levels of unemployment, particularly amongst the youth[4]. Limited employment opportunities have forced many Yemenis to migrate for work to neighbouring countries leaving their families behind. Because of its proximity to conflict affected countries in the Horn of Africa, Yemen is hosting hundreds of thousands of refugees. Yemen also faces one of the largest gender gaps in human development in the world [1] and continues to occupy the last place in the gender gap index rankings of 134 countries and remains the only country in the world to have closed less than 50% of its gender gap[5].

Epidemiologically, the HIV prevalence among the general population of Yemen, including youth is 0.2%[6]. However, high rates of poverty, unemployment, mobility, and gender disparities could place the predominantly young Yemeni population vulnerable to HIV infection. In addition, Yemeni youth, just like other youth in MENA, are experiencing increased premarital sex, peer pressure to engage in risky behaviour, and changing lifestyle norms[7]

Several studies were undertaken in the past few years to understand the vulnerability of Yemeni society to HIV and other sexually transmitted infections (STIs). The four governorate behavioural study[8,9], conducted in 2003 among a sample of participants in their reproductive period (15-49 years) in the governorates of Hodeida, Taiz, Aden, and Hadhramout, generated data on (STIs) awareness and factors predicting vulnerability to HIV infection. The study identified vulnerabilities and risk factors for HIV infection, which included low awareness of the use of condoms, high level of sexually transmitted infections, and unprotected sex. Poverty, a social cultural transition, variations in the sexual subcultures between sections of the Yemeni population, low HIV risk perception and population movements including migration for work, all have emerged as important underlying factors for HIV vulnerability. The vulnerable groups identified included refugees, returnees, youth, truck drivers, Red Sea fishermen, prison inmates, the poorest in the society, and Al-akhdam (Al-akhdam literally means servants in Arabic language, and are a marginalized social group distinct from the majority by their more African features. As a low caste group, they are discriminated against and mostly confined to menial jobs). The study has shown that the main high risk groups are female sex workers and men who have sex with men. The data did not suggest a significant occurrence of injecting drug use[8,9]. In 2008, a bio-behavioural study focusing on 244 female sex workers (FSWs) in Aden has found an HIV prevalence of 1.3% and syphilis prevalence of 4.9%. The majority of FSW (57.1%) reported condom use with their clients, 2.0% reported injecting drugs and 1.2% reported sharing needles and other injecting equipment [6].

Numerous studies have been conducted to explore the knowledge and attitude of young people. A recent comprehensive study on HIV in MENA region has revealed that MENA countries have made considerable gains in increasing HIV/AIDS basic awareness and knowledge in recent years[10]. Comprehensive knowledge, however, including on the use of condoms, is yet far from optimal, and in Yemen, the situation is not much different.

In March-May 2005 a survey among Yemeni high school students in Aden revealed that only 49.4% recognized the protective role of condoms[11]. Stigma and discrimination levels were high among students. Around 40% of the suggested actions by students on how to deal with people living with HIV (PLWH) were punishment actions, which included suggestions for killing PLWH. The results from this study convinced the Education authorities in Aden, Yemen to implement a school-based peer education programme for HIV prevention, which focused on decreasing the level of stigma and discrimination towards people living with HIV and on increasing the knowledge about modes of HIV transmission and prevention. Peer education was selected as the methodology for implementation as it is considered a non-traditional health education method[12], and because the results of the 2005 baseline revealed that schools and friends were the main source of HIV information for students.

Peer Education has a long history; it began being applied in health education and especially for HIV prevention during the 1980s. In recent years, it has been relatively popular in health education, perhaps because of the positive component of the method, which is the interaction it brings between peers[12]. Peer education interventions for HIV/AIDS prevention are usually based on behavioural theories. The design of the intervention discussed here is based partly on the Theory of Diffusion of Innovations [13], which considers that an innovation can be new information, an attitude, a belief or a practice or any other object that is perceived as new by the individual or the community and can be diffused to a specific group. An innovation is communicated through certain channels over time amongst members of a social system (here, the school). A central point in this theory is the use of opinion leaders as ‘change agents'. Peer educators are assumed to have this role by influencing not only those for whom the activities are organized (their peers), but also others of relevance in the peer's environment (family, friends, etc.) through an informal diffusion [14]. Behavioural change thus comes about through a process of formal or informal communication and modelling by trained peers [14]. School-based HIV peer education is a process by which trained students (peer educators) inform, teach and encourage their schoolmates (peers) to recognize the risk factors and protect themselves against HIV. Efforts are made for prevention to become a peer norm.

There is existing research evaluating the efficacy of peer education for HIV prevention in schools, mainly, from countries with concentrated or generalized HIV epidemics, as well as low prevalence countries in Europe [12,15-21]. However, there is limited research evaluating school-based peer education for HIV prevention in cultural and conservative settings such as Yemen, where discussing any issue related to sexuality is difficult, and especially in school settings.

The aim of this evaluation was to assess whether peer education is an effective method for HIV prevention in a high school setting in a culturally conservative Islamic setting. The initial hypothesis was whether the use of peer education methodology could improve knowledge on the modes of prevention and transmission, decrease the level of stigma and discrimination against PLWH, and improve life skills.

## Methods

### The School-Based Intervention

#### Overview

All twenty seven high schools in Aden, Yemen (12 boys' schools, 14 girls' schools, and one mixed school), representing all existing high-schools were the site of the intervention. The intervention included training of school coordinators, selection and training of peer educators, training of school management teams, and implementation at school level.

#### Training of School Coordinators

A team of school coordinators were selected by the education office in Aden, consisting of the director of school supervision, director of school health, director of school social services, a teacher representing the school curriculum supervisory committee, and an expert on training of trainers methodologies. The team was trained in a 9-days workshop (8 hours/day) conducted by 2 international Arabic speaking experts in this field of training of trainers on youth reproductive health, HIV prevention, life skills, and peer education, and the training package was based on a Jordanian life skills and peer education package supported by UNICEF Middle East and North Africa Regional Office[22]. The training package included topics on peer education and training of trainers' methodologies, HIV, reproductive health, sexually transmitted infections, puberty and changes during adolescence, and life skills education. The trained school coordinators team had two functions: coordinating with the schools' managements and parents; and conducting training workshops.

#### Selection and Training of Peer Educators

Peer educators from all 27 high schools were recruited on a voluntarily basis. Among those students volunteered, students were selected who met standard criteria of having good communication skills, being accepted by classmates, and good academic achievement. The selection was done by a committee consisting of the school coordinators and school social workers. The school coordinators visited parents of selected peer educators to explain the programme and to obtain a signed consent form allowing their daughters/sons to participate. Several visits were required, especially to parents of female peer educators, as families are usually reluctant to have their daughters get involved in extra-curricular activities, and especially as these relate to HIV and AIDS prevention. The school coordinators team had a pivotal role in advocating with families. In case of refusal of parents, another peer educator was selected. Parents' refusals mainly occurred in 2005; among the 30 peer educators selected, three parents refused to allow their children to participate, and thus only 27 students were trained as peer educators. However, in the following years, there were no parental refusals, and therefore, during the three years of programme implementation (2005-2008), three refusals were faced among 140 selected peer educators, and thus only 137 peer educators participated in the programme.

The selected peer educators were trained in a 10-days (8 hours/day) workshop. The training was based on the Jordanian life skills and peer education package, in addition to 10 messages on HIV transmission, prevention, and common misconceptions, and 5 life skills messages. HIV messages involved the following: the first message was on the definition of HIV and causative agent; the second on whether we can recognize an infected HIV person by the way he/she looks or do we have to do a blood test, and what does the window period means; the third message was on why HIV is a dangerous infection; the fourth message was on the epidemiology of HIV in Yemen, the middle east and in the world; the fifth was on the mode of transmission; the sixth was on modes of prevention and adopting the ABCD (**a**bstinence, **b**e faithful, use **c**ondoms, and don't use **d**rugs) approach; the seventh was on major misconceptions; the eighth was on risk perception and whether young people can be at risk; the ninth was on how we should deal with PLWH, which stresses the importance of the right of PLWH to live free of stigma and discrimination; and the tenth message was on what is the role of young people in educating the community. The life skills messages were on communication and negotiation skills, self awareness and self-esteem, decision making, respecting difference in opinions, and assertive behaviour.

#### Training of School Management teams

To ensure support for peer educators at school level, school management teams, which consisted of school principals and vice-principals, were trained during a 5-days (7 hours/day) training workshop on peer education methodologies, life skills, and HIV prevention. School coordinators planned with peer educators and management teams the peer activities at their schools.

#### Implementation at School level

Before implementation, a pre-field two-days training was conducted, where peer educators rehearsed the actual messages to be conducted at school level. The ten HIV messages and 2 life skills messages (self-esteem and assertive behaviour) were the major topics covered by peer educators. Peer educators conducted educational sessions as an extra-curricular activity once a week for 90 minutes in a class room setting. Peer education sessions were planned that each student would be targeted with two (90-minutes) sessions in addition to brief sessions during morning school broadcast.

The peer educators used 70 × 50 cm posters, where each message was displayed on one poster. Also, peer education sessions were conducted during youth summer activities in the same class-room style. After the end of the second session, participants received leaflets on the 10 messages, as well as hats and T-shirts with a message: "*Protect yourself with abstinence and knowledge*". The slogan was chosen by the peer educators; the knowledge part referred to the knowledge on condoms and knowledge on avoiding drugs.

#### Magnitude of Intervention

The number of targeted schools was increased gradually, starting in 2005 with the training of 27 peer educators reaching 500 students in 5 schools. Then in 2006 a team of 50 peer educators were trained from 20 schools reached 3,800. In 2007, the existing team of peer educators reached 4,000 students in the same 20 schools, and in 2008, a team of 60 peer educators was established and 1000 students from 27 schools were targeted.

### The Evaluation

#### Design and Participants

A quasi-experimental design for the evaluation of the school-based peer education interventions was applied. Assignment of students to the intervention condition was not based on random sampling, but was based on the time table for extra-curricular activities, where every two weeks around 25 "new" students per school participate in the sessions. The planning of students for extra-curricular activities was indiscriminate with no systematic selection. Randomization was not feasible as it was considered interfering too much with internal school affairs and was considered un-ethical by the implementing as well as funding organizations. The 2005 baseline survey, which was conducted on a randomly selected sample of 2274 students from the same schools, was used as cohort control group

#### Sampling

The sample included in this study consisted of 2510 students selected from 52 randomly selected classes (out of a total of 250 classes) from the 27 high schools located in the 8 districts of Aden governorate. Proportional allocation technique was applied, i.e., class selection was weighted to ensure an appropriate number of classes from each district, dependant on the number of school classes in the district. In each school, the required number of classes was selected by simple random sampling. In the selected classes, all students who were present in the survey day were invited to complete the questionnaire "See additional file 1".

#### Instrument and Data Collection Procedure

A self administered structured questionnaire composed of mainly close-ended questions, and some open questions, was the main study instrument. The questionnaire used was based on the one previously used in the 2005 survey and was subjected to a thorough discussion between the office of education in Aden and active peer educators in high schools of the Aden governorate; the decision was to have the same questionnaire as the one in 2005, and add one more component to study the effects of peer education. Adding behavioural questions on condom use or sexual activity was refused as it was against the policy of the Ministry of Education, due to sensitivities in this conservative school setting. The questionnaire was then pre-tested among one hundred and fifty students from a school not included in the 2005 baseline and this school was excluded from the sample. Pre-testing helped in re-phrasing questions and estimating the approximate time required for completing the questionnaire.

Data collection was conducted during October 2008. The field team consisted of 12 persons subdivided into three smaller teams consisting of a supervisor and three enumerators, each sub-team responsible for almost equal numbers of surveyed classes.

#### Measures

Demographic characteristics that were measured included age and gender. The main study outcome variables included the following:

***General HIV/AIDS awareness- ***represent the assessment of two different variables:

*HIV Infectivity- *was assessed through a closed-ended question on whether HIV is contagious, with options of "yes", "no", and "don't know".

*Existence of HIV/AIDS- *was assessed with two closed-ended questions on whether students thought HIV/AIDS cases existed in Yemen and Aden, with options of "yes", "no", and "don't know"

***Peer Education in Schools***- whether a student was targeted by the intervention or not was assessed by a closed-ended question on whether the student have ever attended/heard of AIDS education by peers in his/her school; yes/no options were available. Those targeted by peer education were then asked through a closed-ended question on whether they found peer education beneficial; beneficial to some extent; or not beneficial.

***Knowledge about HIV/AIDS transmission and prevention***- Students' knowledge regarding HIV/AIDS transmission and prevention were assessed through 12-items. Three options were available: "Yes", "No", and "Don't Know". The answer to each question was scored 1 for correct and 0 for wrong or don't know answer. The total HIV/AIDS transmission and prevention knowledge score was calculated by summing student's responses for the 12 items. The total knowledge score ranged between 0 - 12 and was classified into: Good knowledge (ranging from 9 - 12) representing 75% and above of the maximum point in the score range; Average knowledge (ranging from 6 - 8) representing 50% to less than 75% of the maximum point in the score range; and Poor knowledge (ranging from 0 - 5) representing less than 50% of the maximum point in the score range.

***As an indirect measurement of HIV Stigma and Discrimination, suggested actions towards People Living with HIV- ***was assessed by three open-ended questions, stated as follows: What actions should be taken with persons infected with AIDS by government? What actions should be taken with persons infected with AIDS by society? What actions should be taken with persons infected with AIDS by individuals?

***Life skills Changes- ***students who reported receiving peer education were asked in an open question on whether peer education changed their life skills and if yes, to explain such changes. Answers were coded and categorized into five categories: "No answer", "No change", "There is a change (non-specific)", "There is a specific life-skill change identified", and "Others".

### Data Analysis

Data were analyzed using the Statistical Package for Social Sciences software version 15 (SPSS Incorporation, Chicago, IL, USA). Open ended questions were recorded and coded. Bivariate associations using Pearson Chi-square (χ^2^) were performed for the comparison among proportions. The statistical significance level was set at p-value < .05. The predictive ability of selected independent variables with potential determining relation to the study dependent variables was investigated using multivariate analysis. Knowledge score (0 = poor, 1 = good) and life skills change (0 = not reported, 1 = reported) were dichotomized for binary logistic regression analysis.

Multivariate analysis to explore the effect of the selected independent variables on the likelihood of students to be not receiving peer education, to have good HIV/AIDS knowledge score and to declare life skills changes after receiving HIV/AIDS peer education was performed by binary logistic regression. In this multivariate analysis, model fitness was checked by Hosmer-Lemeshow goodness-of-fit test.

Multi-level analysis was done to study the effect of peer education on the knowledge score, while taking into account the school effect, with knowledge, as an outcome linear variable, and peer education as a predictor variable.

### Ethical Considerations

The Ethics committee of the Faculty of Medicine and Health Science, Sana'a University, granted the ethical approval to conduct this study. In addition, written consent from the Director General of Education Office in Aden was obtained.

## Results

### Characteristics of the participants

Forty-nine percent of the sample (2510 students) was females and fifty-one percent were males. Around 59% were from grade 11 and 41% from grade 12. The majority of students were in the age range 15-17 years (70.4%) with a mean age of 17.06 ± 0.89 years. All students were enrolled in public schools except for one (semi public).

### General HIV/AIDS awareness

Of all students 99.5% (2498 out of 2510) reported that they have heard about AIDS prior to this survey, and among them, 87.7% thought that HIV/AIDS was a disease that existed in Yemen; however a lower proportion (61.0%) perceived its existence in Aden. The majority of the students (80.0%) believed that HIV is infectious.

### Peer Education in Schools

Being targeted by peer education was reported by (78.6%) of students. Among those reported being targeted by peer education, (76.6%) considered peer education beneficial, (21.7%) considered it beneficial to some extent, and only (1.7%) considered it not beneficial.

### Knowledge about HIV/AIDS transmission and prevention

On average, 62.8% of the students had a good score on knowledge about HIV/AIDS transmission and prevention. By their sex, a statistically significant difference was detected (χ^2 ^= (df = 2) = 72.68, p < .01) with 71.2% of the female students having a good knowledge score compared to 54.7% of male students.

Of students targeted by peer education 68% had good knowledge scores, compared with 43.3% of students not targeted by peer education (χ^2 ^= (df = 1) = 111.15, p < .01). Students targeted by peer education had statistically less misconceptions and better knowledge on the modes of transmission and prevention. The only exception was the knowledge on having sex with an infected person, which was not statistically significant (Table 1).

**Table 1:** Knowledge of correct modes of transmission, prevention and misconceptions

	2005		2008		
**Correct modes of transmission and prevention**	**Total** **(N = 2270)** **%**	**Total^∞^** **(N = 2498)** **%**	PE* (N = 1964) **%**	No/PE** (N = 534) **%**	**Significance** p-value*** PE/No PE **2008**

Having sex with infected person can transmit HIV infection	96.2	94.9	95.1	94.4	*NS*

Blood transfusion is a risk factor of HIV infection	93.8	94.7	95.5	91.8	*p *< .01

Shared use of piercing devices with HIV/AIDS patient can transmit HIV	91.6	90.9	92.3	85.8	*p *< .001

Possibility of HIV transmission from infected pregnant mother to her baby	88.0	87.4	88.7	82.4	*p *< .001

Possibility of HIV transmission through breastfeeding from infected mother	71.0	75.5	77.2	69.3	*p *< .001

Male-to-male sex is a risk factor for HIV infection	75.2	70.5	71.8	65.5	*p *< .01

Proper use of male condom is protective against AIDS	49.4	65.4	67.8	56.6	*p *< .001

Possibility of carrying HIV infection by a healthy person	43.0	44.2	47.8	31.1	*p *< .001

**Incorrect modes of transmission and prevention (misconception)**					

Handshaking and kissing can transmit HIV infection	21.6	12.4	10.9	18.0	*p *< .001

Eating and drinking with HIV/AIDS patient can transmit HIV infection	39.7	14.7	13.1	20.8	*p *< .001

Possibility of HIV transmission through swimming pools	31.5	16.1	15.6	17.8	*p *< .001

Possibility of HIV transmission through mosquito bite	49.3	23.8	21.2	33.1	*p *< .001

∞Total of 2008 sample

*Targeted by Peer Education

** Not targeted by Peer Education

***p-value calculated with Pearson's Chi-Squares.

To explore the effect of schools on the knowledge score, a multi level regression analysis was performed. Although this analysis revealed that there was a significant difference between the twenty seven schools in the effect of peer education on knowledge (Wald Z = 4.42, p < .001), the intervention effect of peer education at the individual level was significant. This means that those targeted by peer education had a higher knowledge score (9.24 out of 12.0) compared with those not targeted (7.89 out of 12.0), OR = 2.11, 95% CI = 1.04-4.27, p = .05). Despite the difference between schools, being targeted by peer improved the knowledge of the students regardless of the school they belong to "See additional file 2".

Compared with the cohort control group from the 2005 baseline survey, which included a random sample of 2274 students, those participating in the current study had better knowledge on the modes of transmission and prevention, and less misconceptions. The main difference had been in the knowledge on use of condoms, which increased from 49.4% in 2005 to 67.8% among targeted students in 2008. Misconceptions had decreased, where 21.6% in 2005 reported that transmission through handshaking and kissing can transmit HIV compared with 10.9% in 2008; transmission through eating and drinking was 39.7% in 2005 compared with 13.1%, transmission through swimming pools was 31.5% in 2005 compared with 15.6%, and transmission through mosquitoes was 49.3% in 2005 compared with 21.1% of students targeted with peer education in 2008.

### Suggested actions to deal with People living with HIV/AIDS

We looked at the suggestions of students for actions to be taken towards people living with HIV by the government, society, and individuals. Overall, the most frequent suggested actions were related to Care, Support, and No Discrimination. At government level, the most frequent suggested actions by students were providing medical treatment/specialized hospitals, care and support, and ensuring human rights. For society, most frequent actions included to be treated the same as everyone else by society and avoiding embarrassing/discriminating PLWH. At individual level, the most frequent suggested actions were sympathy/no discrimination and to be treated the same as everyone else by individuals.

We compared the most frequent suggested actions among those targeted and not targeted by peer education. Suggesting care, support and no discrimination actions were statistically associated with being targeted by peer education (Table 2).

**Table 2:** Most frequent suggested actions by school students to deal with people living with HIV

	2005		2008		
**Suggested Actions**	**Total** **(N = 2270)** **%**	**Total^∞^** **(N = 2498)** **%**	PE* (N = 1964) **%**	No/PE** (N = 534) **%**	**Significance** p-value*** (PE/No PE-2008)

Empathy/no discrimination	0	29.9	32.4	20.4	*p *< .001

to be treated the same as everyone else by the society	0	29.9	32.1	21.5	*p *< .001

Treatments/specialized hospitals	7.5	25.1	26.2	21.2	*p *< .05

Avoid embarrassing/discrimination	0	20.1	22.0	13.1	*p *< .001

to be treated the same as everyone else by individuals	0	18.3	20.1	12.0	*p *< .001

Helping and supporting	11.5	15.8	17.0	11.6	*p *< .01

Ensuring human rights	0	14.0	15.3	9.2	*p *< .001

Killing or Whipping	2.4	0	0	0	*-*

Imprisonment	1.9	0	0	0	*-*

∞Total of 2008 sample

*Targeted by Peer Education

** Not targeted by Peer Education

***p-value calculated with Pearson's Chi-Squares.

Compared to the 2005 baseline survey, the most frequent actions suggested by the cohort control group were 2569 suggestions related to punishment and included 158 suggestions for killing PLWH. However, in this study, there were only 321 suggestions for punishment, and all related to isolation, but no suggestions for killing.

### Life Skills Changes and Student Knowledge

Of students targeted by peer education 54% reported life skills changes, with mainly communication skills reported as the main change. Female students reported a statistically higher percentage of life skills changes (65.5%) compared to males (44.5%), (χ2(df = 1) = 87.09, p < .01).

We looked at the relation between reporting life skills changes and knowledge score, and we found a statistically higher percentage of students who had good knowledge score (61.0%) reported life skills changes after peer education activities than those with poor knowledge score (43.9%), (χ^2 ^= (df = 1) = 50.18, p < .01).

When a logistic regression analysis was done, it showed that males (OR = 0.41; 95% CI: 0.34 - 0.50), and those having poor knowledge score (OR = 0.60; 95% CI: 0.49 - 0.74), were at significantly reduced likelihood of changing their life skills after receiving HIV/AIDS peer education.

## Discussion

The school-based peer education interventions implemented in all twenty seven high schools of Aden had succeeded in improving the levels of knowledge of HIV transmission and prevention, and in decreasing the levels of misconceptions, and of stigma and discrimination towards people living with HIV.

The trend over the past several years shows that the level of knowledge on HIV prevention in the Middle East and North Africa (MENA) has increased, although such an improving trend varies among different groups and settings [7]. The findings of this study further confirms this improving trend. One of the important findings of this study is related to the improved knowledge on the use of condoms, which is still considered a sensitive issue to discuss among school students in conservative Muslim and Arab settings [23,24]. Compared with other levels of knowledge of condoms among youth in MENA countries, students in this survey had similar levels of condom knowledge to that in Gaza strip, but had better knowledge than youth in Morroco and Iran [10]. Previous studies in Yemen among school students [10], and among out of school youth[25] had aslo shown lower levels of knowledge on the use of condoms.

The levels of Misconceptions among the students targeted by peer education in this study are lower than other youth surveyed in several MENA countries [10]. In the Islamic Republic of Iran, one-third of high school students believed that HIV can be transmitted by mosquitoes, and 67% out of school youth in Aden believed that HIV can be transmitted by mosquitoes. In addition to decreased levels of misconceptions, there were decreased levels of stigma and discrimination; although the decrease was more apparent among those targeted with peer education, also students not targeted by peer education have suggested far less punishment actions than the cohort control group in 2005. Existing research is also documenting that there is a trend of decreasing stigma and discrimination towards PLWH in MENA, although it is still far from optimal[10].

Although there is ample research exploring HIV knowledge and attitudes in MENA, there is far less on evaluating HIV school interventions among adolescents and young people. In fact, there is only one study, which documents a rapid school peer education intervention, implemented in the United Arab Emirates (UAE), a high income Arab country. In this study, peer education sessions were delivered to male and female students in UAE high schools by college medical students, who were previously trained by a training package developed by international experts[26]. The study revealed that the peer education intervention has improved knowledge and decreased stigma and discrimination among the students who received the intervention.

The relatively new aspect about this study, in addition to being implemented in the most traditional and disadvantaged country of the MENA region [1], it documents a three-year school-based peer education as well as a life skills intervention, where high school male and female adolescents were trained as peer educators and led the process in their own schools. Moreover, students were given messages on the protective role of condoms. This is noteworthy in such a conservative culture, where adolescents are not considered to have the right to information related to sexuality. The acceptance of the intervention, especially by families of peer educators is an important finding. The fact that there were only three refusals from parents, reflect the important advocacy work by the school programme coordinators. Although the conservative nature of Arab and Muslim societies had usually been blamed for not implementing school-based interventions on HIV and sexual health, this study indicates that such interventions in conservative settings are possible, and parents could be willing to accept the participation of their adolescent girls, given that they are well informed by schools.

The school-based peer education has increased not only the overall knowledge on condoms for HIV prevention compared with the school baseline survey, but has also decreased the knowledge gender gap on condoms, which was evident in the baseline survey conducted in 2005[11]. The gender gap favouring males, regarding the knowledge on condoms, was also huge among young people in vulnerable communities of Aden, which was revealed by the study conducted by Al-Serouri et al in 2005[25]. This gender difference is a reflection of the traditional Yemeni society, which gives young men more freedom than young women to discuss sexual related matters, such as condom use [25]. However, the result of this current study challenge this fact, where female students attained better knowledge on condoms after being targeted by peer education, compared to male students. They also reported more life skill changes than male students. This suggests that the design of the intervention was gender sensitive such that it was able to reach female students with the necessary information and skills. It also indicates that girls in Yemen make maximum benefit of all existing educational opportunities, and that girl's education is the most important determinant factor that can lead to gender equality, in a nation with huge gender disparities [5]. Previous research [26] has also documented that female students had attained better knowledge than their male counterparts after participating in HIV peer education in schools, although female students had lower levels of knowledge at baseline.

Although some might argue that improving levels of knowledge and decreasing levels of stigma and discrimination do not necessarily lead to behavioural change, it should be noted that reporting life skills changes was associated with a good knowledge score, which indicates that the peer education intervention not only contributed to knowledge on HIV and decreasing stigma and discrimination, but also to improving life skills, which is an integral part of adopting safe sexual behaviour [27].

Several limitations are related to this study. The first limitation is due to the fact that this study evaluated an intervention which aimed to improve knowledge and decrease stigma and discrimination but did not measure the impact on sexual behaviour. Unfortunately, this was not possible due to the conservative school system and the cultural setting. Previous evaluations performed in developing countries have indicated that peer education contributed to knowledge and awareness, but had weak to moderate effects on adolescents' sexual risk behaviour [28-31]. Other studies have shown that peer-led programs improved students' attitudes and behaviours compared with students who did not receive such prevention education [18,32,33]. However, in a setting like Yemen, where access to information by adolescents is still limited, increase in knowledge and awareness is a crucial step towards behaviour change, as one of the factors that influence HIV risk behaviour among young people is their HIV/AIDS knowledge [34].

The second limitation is related to the quasi-experimental design of the study, where internal validity is a concern because of the lack of randomization among intervention and control groups. However, several measures were undertaken to improve the similarities between cases and controls. The fact that students from the same schools were cases and controls decreased the non-equivalency between them. Also, the 2005 baseline was used as a cohort control group, which helped to increase the internal validity of the study [35].

The third limitation is related to the external validity and generalizability of the study. The fact that this peer education intervention was conducted in Aden governorate does not necessarily mean that the findings can be generalized to all of Yemen. Aden governorate is an urban setting, while 75% of the Yemeni population is living in rural areas [3]. The fourth limitation is related to the validity and reliability of studies that use structured quantitative tools, where young people might give the desirable answers regarding skills and attitudes. The fifth limitation is related to the fact that the significant change in knowledge and attitudes among students may in part reflect changes over time, rather due to the peer education intervention.

Although this study has all of the above detailed limitations, it has revealed that it is possible to address HIV, a sensitive issue related to sexuality, in schools of a conservative country like Yemen, and these findings could be a starting point for future school-based sexual and reproductive health programmes in Yemeni schools. Such programmes should be life skills based and culturally sensitive and should be integrated within the Yemeni school curriculum as a long term strategy. This integration requires policy development by the Ministry of Education and the gap in the short term should be filled through integration in extra-curricular activities. It is also recommended to conduct a youth risk assessment survey, which would be a valuable tool in the context of Yemen, where there is much more to learn about the risks and vulnerabilities associated with adolescents and young people in Yemen. Such a survey, although research tools are available from other countries, should be tailored to the Yemeni context. The survey should not only focus on youth vulnerabilities and risks to HIV, but also sexually transmitted infections, alcohol abuse, and Qat (Khat) consumption. Khat, a green shrub, which is a mild narcotic with amphetamine-like effects, is widely chewed by Yemeni youth. Previous research had conflicting conclusions on whether Khat could be a risk factor for HIV and other sexually transmitted infections [36,37], and thus this issue has to be further explored within the context of a youth risk assessment survey.

## Conclusion

This evaluation demonstrated that HIV education among school adolescents is possible in very conservative settings, given the interventions are addressed in a culturally sensitive manner, and all key stakeholders are involved in the early stages of the interventions. The school-based peer education intervention has succeeded in improving levels of knowledge on modes of transmission and prevention, and in decreasing levels of stigma and discrimination.

## Competing interests

The authors declare that they have no competing interests.

## Authors' contributions

BA - conceived and designed the study; supervised the implementation of the interventions during 2005-2008; and drafted the manuscript. HB and KA - supervised field data collection and were significantly involved in the data entry and analysis. RC - assisted with the data analysis and revised the article. BvdB and GK - were significantly involved in interpretation of data, in revising the article critically for important intellectual content. All authors read and approved the final manuscript.

## Pre-publication history

The pre-publication history for this paper can be accessed here:

http://www.biomedcentral.com/1471-2458/11/279/prepub

## Supplementary Material

Additional file 1 **Sampling Details**. Details on the sampling process is provided. Two tables are included, which describe the distribution of grade 11 and 12 students in the different districts, and the distribution of sampled classes by schools in the eight districts of Aden Governorate. Click here for file

Additional file 2 **Differences between schools in relation to knowledge among students targeted and not targeted by peer education**. The file includes SPSS output of multi-level regression analysis. The analysis reveals that although there was a significant difference among schools, the intervention effect of peer education at the individual level was significant. Click here for file
