# Supplementary material for: Evaluation of a school-based HIV prevention intervention among Yemeni adolescents
Source: BMC Public Health. 2011 May 7;11:279. doi: 10.1186/1471-2458-11-279 (PMC3112119; doi:10.1186/1471-2458-11-279)
Supplement: Additional file 1 — Sampling Details. Details on the sampling process is provided. Two tables are included, which describe the distribution of grade 11 and 12 students in the different districts, and the distribution of sampled classes by schools in the eight districts of Aden Governorate. [file 1471-2458-11-279-S1.DOCX]

**Additional file 1: Sampling Details**

The sample included in this study consisted of 2510 students, which was around twenty percent of the total number of grade 11 and 12 high school students (12,269) in Aden governorate were targeted in this study. According to the details provided by the office of education in Aden governorate the mean number of students in each class was 49 students with a total of 250 classes with grade 11 and 12 students. Thus the total number of classes for the sample was 51 (2510/49). The required number of classes in each district and schools was selected using the proportional allocation technique. In each school, the required number of classes was selected by simple random sampling. In the selected classes, all students who were present in the survey day were invited to complete the questionnaire. For instance, the total number of classes of grade 11 and 12 in the two secondary schools in Maalla district is 22 which is amounted to 8.8% of the total number of classes (22 out of 250). This means 8.8% of the total number of the required classes is needed from Maalla. Therefore, four classes were enrolled from Maalla (4 is 8.8% of 50). In each school, the required number of classes was selected by simple random sampling. In the example of Maalla, nine of the 22 classes are found in the girls’ school whereas 13 in the boys’ school. Therefore, two classes (one class from grade 11 and one from grade 12) were randomly selected from each school to attain the four required classes.

**Distribution of grade 11 and 12 students in the different districts**

| **District** | **Grade 12 Classes** | **Grade 12 Students** | **Grade 11 Classes** | **Grade 11 Students** | **Total students** | **No. of Classes** | **% of classes** | **Required no. of Classes** |
| --- | --- | --- | --- | --- | --- | --- | --- | --- |
| Maalla | 11 | 550 | 11 | 532 | 1082 | 22 | 8.8 | **4** |
| Al-Tawahi | 13 | 526 | 12 | 553 | 1079 | 25 | 10.0 | **5** |
| Khor- Makser | 10 | 539 | 9 | 575 | 1114 | 19 | 7.6 | **4** |
| Sirah | 19 | 883 | 21 | 906 | 1789 | 40 | 15.9 | **8** |
| Al-Buraiqa | 14 | 641 | 14 | 694 | 1335 | 28 | 11.2 | **6** |
| Sheikh Othman | 21 | 1087 | 22 | 1097 | 2184 | 43 | 17.1 | **9** |
| Dar- Sa'ad | 16 | 792 | 19 | 966 | 1758 | 35 | 13.9 | **7** |
| Mansoura | 17 | 877 | 21 | 1051 | 1928 | 38 | 15.2 | **8** |
| **Total** | **121** | **5895** | **129** | **6374** | **12269** | **250** | **100** | **51** |

Source: Aden Education Office. Secondary School Statistics in Aden (2007-2008)

Source: Education Office, Aden. Secondary School Students’ Statistics in Aden (2006-2007)

**Distribution of sampled classes by schools in the eight districts of Aden Governorate**

| **School** | **Classes** |
| --- | --- |
| 1. **Maalla District (4 classes)** | |
| Mareb | 2 |
| 14th October | 2 |
| 1. **Al-Tawahi District (5 classes)** | |
| Muhrez | 1 |
| Garadah | 2 |
| Tamna'a | 2 |
| 1. **Khor Makser District (4 classes)** | |
| Al-Dawliah Al-Shamilah | 1 |
| Mohammed Abdo Ghanem | 1 |
| Abdo Albary Kasem | 2 |
| 1. **Sirah District (8 classes)** | |
| Lutfi Gaafer Aman | 3 |
| Aban | 2 |
| Bakatheer | 2 |
| Al-Baihani | 1 |
| 1. **Al-Buraiqa District (6 classes)** | |
| Mohammed Al-Durah | 1 |
| Salah Addeen | 1 |
| Al-Wahda | 1 |
| Nasser Rashed Luta | 1 |
| Al-Sha'ab | 1 |
| Al-Quds | 1 |
| 1. **Sheikh Othman (9 classes)** | |
| Othman Abdo | 3 |
| Belqees | 4 |
| Al-Nahda | 2 |
| 1. **Dar Sa’ad District (7 classes)** | |
| Zainab Ali Kasem | 3 |
| Aden | 1 |
| Al-Ahdel | 3 |
| 1. **Al-Mansoura District (8 classes)** | |
| Khadija | 2 |
| Al-Numan | 4 |
| Ba-Dib | 2 |
| **Grand Total** | **51** |
