# Supplementary material for: Evaluation of a school-based HIV prevention intervention among Yemeni adolescents
Source: BMC Public Health. 2011 May 7;11:279. doi: 10.1186/1471-2458-11-279 (PMC3112119; doi:10.1186/1471-2458-11-279)
Supplement: Additional file 2 — Differences between schools in relation to knowledge among students targeted and not targeted by peer education. The file includes SPSS output of multi-level regression analysis. The analysis reveals that although there was a significant difference among schools, the intervention effect of peer education at the individual level was significant. [file 1471-2458-11-279-S2.DOC]

Additional file 2: Differences between schools in relation to knowledge among students targeted and not targeted by peer education.

| Schools | Peer Education | Count | Knowledge Mean | Standard Deviation | Coefficient of Variation |
| --- | --- | --- | --- | --- | --- |
| Garadeh | yes | 37 | 8.11 | 1.853 | 22.8% |
|  | no | 39 | 6.59 | 2.302 | 34.9% |
|  | Total | 76 | 7.33 | 2.217 | 30.3% |
| Tamna’a | yes | 48 | 7.79 | 2.103 | 27.0% |
|  | no | 15 | 7.53 | 1.767 | 23.5% |
|  | Total | 63 | 7.73 | 2.018 | 26.1% |
| Muhairez | yes | 37 | 9.86 | 1.228 | 12.5% |
|  | no | 13 | 8.00 | 2.944 | 36.8% |
|  | Total | 50 | 9.38 | 1.978 | 21.1% |
| Moh’d Abdo Ghanem | yes | 31 | 7.90 | 1.868 | 23.6% |
|  | no | 34 | 6.82 | 2.249 | 33.0% |
|  | Total | 65 | 7.34 | 2.131 | 29.0% |
| Abdulbari | yes | 62 | 6.79 | 2.097 | 30.9% |
|  | no | 37 | 6.16 | 2.102 | 34.1% |
|  | Total | 99 | 6.56 | 2.110 | 32.2% |
| Al Dawliah | yes | 39 | 7.95 | 2.339 | 29.4% |
|  | no | 4 | 7.50 | 1.291 | 17.2% |
|  | Total | 43 | 7.91 | 2.255 | 28.5% |
| Mareb | yes | 72 | 8.10 | 1.855 | 22.9% |
|  | no | 28 | 7.86 | 1.693 | 21.6% |
|  | Total | 100 | 8.03 | 1.806 | 22.5% |
| 14October | yes | 99 | 10.62 | 1.383 | 13.0% |
|  | no | 9 | 8.33 | 1.936 | 23.2% |
|  | Total | 108 | 10.43 | 1.560 | 15.0% |
| Bakatheer | yes | 61 | 7.98 | 2.053 | 25.7% |
|  | no | 7 | 6.71 | 2.360 | 35.2% |
|  | Total | 68 | 7.85 | 2.104 | 26.8% |
| Aban | yes | 60 | 9.77 | 1.661 | 17.0% |
|  | no | 6 | 9.83 | 1.602 | 16.3% |
|  | Total | 66 | 9.77 | 1.644 | 16.8% |
| Lutfi | yes | 106 | 8.89 | 2.108 | 23.7% |
|  | no | 30 | 7.63 | 2.157 | 28.3% |
|  | Total | 136 | 8.61 | 2.175 | 25.3% |
| Al Baihani | yes | 43 | 10.79 | 1.081 | 10.0% |
|  | no | 2 | 11.00 | .000 | .0% |
|  | Total | 45 | 10.80 | 1.057 | 9.8% |
| Al Numan | yes | 162 | 9.83 | 1.667 | 17.0% |
|  | no | 24 | 8.88 | 1.727 | 19.5% |
|  | Total | 186 | 9.70 | 1.700 | 17.5% |
| Batheeb | yes | 75 | 10.60 | 1.952 | 18.4% |
|  | no | 9 | 11.44 | .726 | 6.3% |
|  | Total | 84 | 10.69 | 1.875 | 17.5% |
| Khadijah | yes | 79 | 10.09 | 2.231 | 22.1% |
|  | no | 14 | 10.93 | .730 | 6.7% |
|  | Total | 93 | 10.22 | 2.095 | 20.5% |
| Othman | yes | 124 | 9.31 | 2.108 | 22.6% |
|  | no | 47 | 8.02 | 2.715 | 33.8% |
|  | Total | 171 | 8.95 | 2.354 | 26.3% |
| Al-Nahdah | yes | 73 | 8.86 | 1.805 | 20.4% |
|  | no | 45 | 7.78 | 2.163 | 27.8% |
|  | Total | 118 | 8.45 | 2.011 | 23.8% |
| Belqees | yes | 127 | 9.39 | 2.020 | 21.5% |
|  | no | 34 | 9.44 | 1.829 | 19.4% |
|  | Total | 161 | 9.40 | 1.976 | 21.0% |
| Zainab | yes | 155 | 10.13 | 1.467 | 14.5% |
|  | no | 10 | 10.10 | 1.370 | 13.6% |
|  | Total | 165 | 10.13 | 1.457 | 14.4% |
| Aden | yes | 45 | 10.38 | 1.267 | 12.2% |
|  | no | 3 | 11.00 | 1.000 | 9.1% |
|  | Total | 48 | 10.42 | 1.252 | 12.0% |
| Al-Ahdel | yes | 151 | 7.77 | 2.501 | 32.2% |
|  | no | 60 | 6.90 | 2.529 | 36.7% |
|  | Total | 211 | 7.53 | 2.534 | 33.7% |
| Mohamed Durrah | yes | 70 | 10.00 | 1.523 | 15.2% |
|  | no | 4 | 8.75 | .957 | 10.9% |
|  | Total | 74 | 9.93 | 1.520 | 15.3% |
| Naser Rasheed Lutah | yes | 40 | 8.28 | 2.353 | 28.4% |
|  | no | 15 | 7.53 | 1.407 | 18.7% |
|  | Total | 55 | 8.07 | 2.150 | 26.6% |
| Al Wehdah | yes | 34 | 8.09 | 2.503 | 30.9% |
|  | no | 21 | 7.29 | 2.101 | 28.8% |
|  | Total | 55 | 7.78 | 2.370 | 30.5% |
| Salah Adeen | yes | 43 | 9.35 | 2.126 | 22.7% |
|  | no | 5 | 9.40 | 1.949 | 20.7% |
|  | Total | 48 | 9.35 | 2.088 | 22.3% |
| Al Shaab | yes | 41 | 10.32 | 1.540 | 14.9% |
|  | no | 10 | 10.60 | .843 | 8.0% |
|  | Total | 51 | 10.37 | 1.428 | 13.8% |
| Alqudus | yes | 50 | 9.88 | 1.560 | 15.8% |
|  | no | 9 | 8.56 | 1.944 | 22.7% |
|  | Total | 59 | 9.68 | 1.676 | 17.3% |
| Total | **yes** | **1964** | **9.24** | **2.165** | **23.4%** |
|  | **no** | **534** | **7.89** | **2.424** | **30.7%** |
|  | **Total** | **2498** | **8.95** | **2.290** | **25.6%** |

**Estimates of Covariance Parameters(a)**

| **Parameter** | | **Estimate** | **Std. Error** | **Wald Z** | **Sig.** | **95% Confidence Interval** | |
| --- | --- | --- | --- | --- | --- | --- | --- |
|  |  |  |  |  |  | **Lower Bound** | **Upper Bound** |
| Residual | | 3.823924 | .109424 | 34.946 | .000 | 3.615360 | 4.044519 |
| q16 [subject = q1] | Variance | 1.459524 | .330034 | 4.422 | .000 | .936988 | 2.273467 |

a Dependent Variable: Total knowledge of transmission & prevention.
